# Dermatoses in Pregnancy: A Hospital-Based Cross-Sectional Study From Eastern India

**DOI:** 10.7759/cureus.110780

**Published:** 2026-06-13

**Authors:** Doyir Bagra, Debabrata Ghosh, Sudipta Biswas, Koushik Biswas, Kaushik Shome, Joly Seth

**Affiliations:** 1 Dermatology, Venereology and Leprosy, Burdwan Medical College, Burdwan, IND; 2 Biochemistry, All India Institute of Medical Sciences, Raebareli, IND

**Keywords:** dermatoses, india, physiological changes, pregnancy, pregnant females, skin lesions, specific dermatoses of pregnancy

## Abstract

Introduction

Numerous skin changes occur in pregnancy, the majority of which are physiological in nature and linked to the hormonal, metabolic, vascular, or immunologic changes. However, some pruritic eruptions cause anxiety during pregnancy and may affect the maternal and fetal outcome. This study aims to evaluate the prevalence, clinical patterns, and association with gravida and trimester of dermatoses among pregnant women attending an antenatal clinic for routine obstetric care.

Methods

This descriptive cross-sectional study was conducted at the Department of Dermatology, Venereology and Leprosy in collaboration with the Department of Obstetrics and Gynecology of Burdwan Medical College and Hospital, Burdwan, India. A total of 138 pregnant women attending the Outpatient Department of both these specialties, from August 2023 to July 2025, were included. Patients were evaluated by detailed history taking and clinical examination at the time of presentation. Suspected cases of bacterial, fungal, or viral infection were confirmed by laboratory diagnosis. The data were analyzed using SPSS version 24 (IBM Corp., Armonk, NY).

Results

Among the 138 pregnant women, physiological skin changes were observed in 136 (98.5 %), with pigmentary changes being more frequent (129, 93.4 %). Specific dermatoses of pregnancy were observed in 34 (24.6 %), with prurigo of pregnancy being the most common (n = 17, 12.31%). Out of 30 (21.7%) women affected by infectious diseases, fungal infection was the most common (n = 20, 14.4%). The primigravida had significantly higher pigmentary changes (p = 0.0265) and striae gravidarum (p = 0.012). Specific dermatoses of pregnancy were significantly higher in the third trimester (p < 0.001).

Conclusion

Physiological skin changes, which are more common than specific dermatoses during pregnancy, require only reassurance. However, careful observation and examination are necessary as some dermatological conditions are affected by pregnancy.

## Introduction

Pregnancy is a unique physiological state characterized by profound hormonal, immunological, and metabolic changes that significantly influence various organ systems, including the skin. As the most visible organ, the skin often reflects these internal physiological alterations, manifesting in a wide spectrum of dermatological conditions [1]. Skin changes during pregnancy may be physiological changes, pregnancy-specific dermatoses, or dermatoses influenced by pregnancy. A clear understanding of these conditions is essential for clinicians to differentiate benign conditions from a potentially harmful disorder, thereby ensuring optimal maternal and fetal outcomes [2].

Physiological skin changes during pregnancy are common and can be classified into pigmentary changes, vascular changes, glandular changes, and connective tissue alterations. These changes are generally benign and self-limiting, which resolve in the postpartum period, although they may cause cosmetic concern and psychological distress [3]. In contrast, certain dermatoses are unique to pregnancy and might carry significant risks. These include pruritic urticarial papules and plaques of pregnancy (PUPPP), intrahepatic cholestasis of pregnancy (ICP), pemphigoid gestationis, and atopic eruption of pregnancy. Early recognition and appropriate management of these conditions are crucial to prevent adverse maternal and fetal outcomes [4]. Dermatoses influenced by pregnancy are pre-existing skin conditions like acne, systemic lupus erythematosus (SLE) rashes, atopic dermatitis, psoriasis, and infections like candidiasis, whose course changes during pregnancy due to hormonal and immunological shifts. These might worsen, improve, or remain unchanged [5].

PUPPP is one of the most common pregnancy-specific dermatoses, typically presenting in the third trimester with intensely pruritic erythematous papules and plaques involving abdominal striae and sparing the periumbilical area. Although benign and self-limiting, it can significantly impair quality of life [6]. Pemphigoid gestationis, a rare autoimmune blistering disorder, usually occurs in the second or third trimester and may be associated with preterm delivery and low birth weight [7]. Atopic eruption of pregnancy, including eczema, prurigo, and pruritic folliculitis, often presents earlier in pregnancy. It includes flares of atopic dermatitis or new onset atopy and does not pose a significant risk to the fetus [8]. ICP is characterized by generalized pruritus without primary skin lesions and is associated with serious fetal complications, including preterm birth and stillbirth [9].

Pregnancy can also influence the course of pre-existing dermatological conditions such as psoriasis, atopic dermatitis, and acne [10]. While some conditions may improve due to the immunomodulatory state of pregnancy, others may worsen and require careful therapeutic planning to ensure maternal comfort while minimizing fetal risk [11]. Management of dermatoses in pregnancy requires a multidisciplinary approach involving dermatologists and obstetricians. Therapeutic options must be selected judiciously, with preference given to topical agents over systemic treatments whenever possible, to alleviate maternal symptoms while ensuring fetal safety [12].

Benign conditions like atopic eruption of pregnancy and polymorphic eruption of pregnancy are self-limiting and typically respond to topical moisturizer (emollients), moderate potency corticosteroids (mometasone, fluticasone), antihistamine (chlorpheniramine, loratadine, and cetirizine) [13]. Pruritic folliculitis is mild and managed with topical benzoyl peroxide or narrowband ultraviolet B (UVB) phototherapy [14,15]. Pemphigoid gestationis is initially treated with systemic corticosteroids (prednisolone 0.5 mg/kg/day, for a few days); refractory cases require intravenous immunoglobulin (IVIG) [16,17]. Ursodeoxycholic acid remains the treatment of choice for intrahepatic cholestasis [18]. Dermatological disorders during pregnancy can significantly affect the mental health of the pregnant woman, which can risk-averse pregnancy outcomes. There is a paucity of data on the epidemiology of dermatoses among pregnant women attending from Eastern India. This study aims to evaluate the prevalence, clinical patterns, and association with gravida and trimester of dermatoses among pregnant women attending an antenatal clinic for routine obstetric care.

## Materials and methods

Study design

The descriptive cross-sectional study was conducted at the Department of Dermatology, Venereology and Leprosy in collaboration with the Department of Obstetrics and Gynecology of Burdwan Medical College and Hospital, Burdwan, a tertiary care center in Eastern India. The study was carried out from August 2023 to July 2025. Approval from the Institutional Ethics Committee was obtained before starting the study (memo no. BMC/IEC/226 dated 17.07.2023). Written informed consent was obtained from all participants in the study. Throughout the study, patient confidentiality was maintained.

Study participants

The study included all pregnant women, irrespective of age and gravidity, who attended the Dermatology Outpatient Department (OPD) and pregnant women with skin lesions attending the Antenatal Clinic of the Department of Obstetrics and Gynecology for routine check-up. All pregnant women attending the Antenatal Clinic were systematically screened for dermatological manifestations irrespective of the presence or absence of skin complaints. There was a referral system for transferring pregnant women with non-physiological dermatological complaints from the Antenatal Clinic to the Dermatology OPD. Only those who provided written informed consent for the study were included. Out of 138 participants, 75 were recruited from the Dermatology OPD and 63 from the Antenatal Clinic. Pregnant women who had conceived through in vitro fertilization and those who were admitted to the Inpatient Department (IPD) Antenatal ward of the Obstetrics and Gynecology department or Critical Care Unit were excluded from the study. Consecutive recruitment was ensured by selecting every eligible subject who met the study's inclusion and exclusion criteria during the study period.

Sample size

The sample size was calculated using Cochran's formula, $n = \frac{Z^{2}p(1 - p)}{d^{2}}$, where n is the required sample size, z is the standard normal deviation at a 95% confidence level (1.96), p is the prevalence, and d is the error margin at 5% (0.05 standard value). Based on a study conducted by Kar et al. [19], which reported the prevalence of dermatoses in pregnancy of 90%, and substituting the value, z = 1.96, p = 0.9, and d = 0.05, in the formula, the calculated sample size was 138. Accordingly, a total of 138 patients were included in this study.

Study procedure

All patients were evaluated through detailed history taking and clinical examination at the time of presentation. Information regarding gestational age, trimester, gravidity, and dermatological complaints was recorded. Based on this assessment, patients were categorized into physiological changes in pregnancy, specific dermatoses of pregnancy, and non-specific dermatoses of pregnancy as described previously by Kannambal and Tharini [20].

For identification of physiological changes in pregnancy, a complete examination of the skin and appendages was performed. In cases where specific dermatoses of pregnancy were suspected, the morphology, distribution, and extent of the lesion were carefully assessed. The diagnostic criteria used for prurigo of pregnancy, PUPPP, pruritic folliculitis, and pruritus gravidarum were based on internationally accepted classification systems as mentioned by Ambros-Rudolph et al. [12]. Systemic examination was performed wherever required. In addition, non-specific dermatoses occurring during pregnancy were identified and recorded. 

In patients with suspected infections, appropriate investigations were carried out, including potassium hydroxide (KOH) mount for fungal infections, Gram stain for bacterial infection, and Tzanck smear in vesicular lesions to look for features suggestive of viral etiology. Those presenting with generalized pruritus, especially in the absence of primary skin lesions, were further evaluated with liver function tests to exclude ICP. In cases where clinical diagnosis was uncertain, additional investigations, such as biopsy, were performed. Direct immunofluorescence was performed in selected cases where an autoimmune blistering disorder was suspected. Routine investigations, including complete blood count and blood glucose level, were performed where indicated. As per the national guidelines, all pregnant women underwent screening for sexually transmitted infections, including syphilis by Venereal Disease Research Laboratory (VDRL) test and human immunodeficiency virus (HIV) by enzyme-linked immunosorbent assay (ELISA).

Data collection and statistical analysis

Data collected from history, clinical examination, and relevant investigation were entered into a Microsoft Excel sheet (Microsoft Corporation, Redmond, WA). The data were analyzed using SPSS version 24 (IBM Corp., Armonk, NY). Categorical variables like physiological changes, different types of pregnancy-specific dermatoses, and their distribution across gravidity and trimester were expressed as frequency and percentage. The association between categorical variables with trimester and gravidity was analyzed using the chi-square test or Fisher’s exact test, as appropriate. A p-value of less than 0.05 was considered statistically significant.

## Results

A total of 138 pregnant women were included in the study. The mean age of the participants was 24.6 ± 3.5 years. Their age ranged from 18 to 38 years. Eighty-six (62.3%) participants were primigravida, followed by 24 (17.4%) in second gravida, 20 (14.5%) in third gravida, and eight (5.8%) in fourth gravida. With respect to pregnancy trimester, 76 (55.1%) participants were in their third trimester, followed by 56 (40.6%) in their second trimester and six (4.3%) in their first trimester at the time of study.

We observed physiological cutaneous changes among 136 (98.5%) participants. Pigmentary changes were the most common physiological changes, observed in 129 (93.4%) participants. Among them, linea nigra was the most common finding, seen in 118 (85.5%) participants, followed by secondary areola in 103 (74.6%) participants and melasma in 58 (42.0%) participants. Connective tissue changes were noted in 104 (75.3%) participants. Among the connective tissue changes, striae gravidarum was the most common finding, being observed in 104 (75.4%) participants, while acrochordon was observed in three (2.17%) participants. Vascular changes were present in 30 (21.7%) participants. Pedal edema was the most common vascular change, occurring in 24 (17.4%) participants. Other vascular changes, such as vulvar edema, spider nevi, telangiectasia, and pyogenic granuloma, occurred in four (2.8%), two (1.4%), two (1.4%), and one (0.72%) participants, respectively. Eccrine gland changes, hair changes, and nail changes were observed in five (3.6%), five (3.6%), and three (2.2%) participants, respectively. The detailed distribution of physiological changes in pregnancy, trimester-wise and gravida-wise, is presented in Table 1.

**Table 1:** Distribution of physiological skin changes in pregnancy n: absolute count, %: percentage, G1: primigravida, G2: second gravida, G3: third gravida, G4: fourth gravida

	First trimester				Second trimester				Third trimester				n (%)
	G1	G2	G3	G4	G1	G2	G3	G4	G1	G2	G3	G4	
	n (%)	n (%)	n (%)	n (%)	n (%)	n (%)	n (%)	n (%)	n (%)	n (%)	n (%)	n (%)	
Pigmentation													129 (93.4%)
Linea nigra	2 (1.4%)	1 (0.7%)	0 (0.0 %)	0 (0.0%)	35 (25.4%)	9 (6.5%)	8 (5.8%)	2 (1.4%)	41 (29.7%)	10 (7.2%)	8 (5.8%)	2 (1.4%)	118 (85.5%)
Secondary areola	3 (2.2%)	2 (1.4%)	0 (0.0%)	0 (0.0%)	27 (19.6%)	8 (5.8%)	3 (2.2%)	3 (2.2%)	39 (28.3%)	8 (5.8%)	5 (3.6%)	5 (3.6%)	103 (74.6%)
Melasma	1 (0.7%)	1 (0.7%)	0 (0.0%)	0 (0.0%)	14 (10.1%)	6 (4.3%)	2 (1.4%)	1 (0.7%)	22 (15.9%)	8 (5.8%)	2 (1.4%)	1 (0.7%)	58 (42.0%)
Vascular													30 (21.7%)
Pedal edema	2 (1.4%)	0 (0.0%)	0 (0.0%)	0 (0.0%)	0 (0.0%)	0 (0.0%)	0 (0.0%)	1 (0.7%)	2 (1.4%)	5 (3.6%)	10 (7.2%)	4 (2.8%)	24 (17.4%)
Vulvar edema	0 (0.0%)	0 (0.0%)	0 (0.0%)	0 (0.0%)	0 (0.0%)	0 (0.0%)	0 (0.0%)	1 (0.7%)	0 (0.0%)	1 (0.7%)	1 (0.7%)	1 (0.7%)	4 (2.8%)
Spider nevus	0 (0.0%)	0 (0.0%)	0 (0.0%)	0 (0.0%)	0 (0.0%)	0 (0.0%)	0 (0.0%)	1 (0.7%)	0 (0.0%)	0 (0.0%)	1 (0.7%)	0 (0.0%)	2 (1.4%)
Telangiectasia	0 (0.0%)	0 (0.0%)	0 (0.0%)	0 (0.0%)	0 (0.0%)	0 (0.0%)	1 (0.7%)	0 (0.0%)	0 (0.0%)	0 (0.0%)	0 (0.0%)	1 (0.7%)	2 (1.4%)
Pyogenic granuloma	0 (0.0%)	0 (0.0%)	0 (0.0%)	0 (0.0%)	0 (0.0%)	0 (0.0%)	0 (0.0%)	0 (0.0%)	0 (0.0%)	0 (0.0%)	1 (0.7%)	0 (0.0%)	1 (0.7%)
Connective tissue changes													104 (75.4%)
Striae gravidarum	2 (1.4%)	1 (0.7%)	0 (0.0%)	0 (0.0%)	21 (15.2%)	6 (4.3%)	2 (1.4%)	1 (0.7%)	48 (34.8%)	12 (8.7%)	8 (5.8%)	3 (2.2%)	104 (75.4%)
Acrochordon	0 (0.0%)	0 (0.0%)	0 (0.0%)	0 (0.0%)	0 (0.0%)	0 (0.0%)	1 (0.7%)	0 (0.0%)	0 (0.0%)	0 (0.0%)	1 (0.7%)	1 (0.7%)	3 (2.2%)
Eccrine changes													5 (3.6 %)
Miliaria	0 (0.0%)	0 (0.0%)	0 (0.0%)	0 (0.0%)	1 (0.7%)	1 (0.7%)	0 (0.0%)	0 (0.0%)	0 (0.0%)	1 (0.7%)	2 (1.4%)	0 (0.0%)	5 (3.6 %)
Nail changes	0 (0.0%)	0 (0.0%)	0 (0.0%)	0 (0.0%)	1 (0.7%)	0 (0.0%)	0 (0.0%)	0 (0.0%)	2 (1.4%)	0 (0.0%)	0 (0.0%)	0 (0.0%)	3 (2.2 %)
Hair changes													5 (3.6 %)
Hirsutism	0 (0.0%)	0 (0.0%)	0 (0.0%)	0 (0.0%)	0 (0.0%)	1 (0.7%)	0 (0.0%)	0 (0.0%)	0 (0.0%)	1 (0.7%)	1 (0.7%)	0 (0.0%)	3 (2.2 %)
Hair loss	0 (0.0%)	0 (0.0%)	0 (0.0%)	0 (0.0%)	0 (0.0%)	0 (0.0%)	0 (0.0%)	0 (0.0%)	0 (0.0%)	1 (0.7 %)	1 (0.7%)	0 (0.0%)	2 (1.4 %)
Total													136 (98.5 %)

Specific dermatoses of pregnancy were observed in 34 (24.6%) participants. Prurigo of pregnancy was the most common disorder, affecting 17 (12.3%) participants, followed by PUPPP in 11 (7.9%), pruritus gravidarum in six (4.3%), and pruritic folliculitis in two (1.4%) participants. No cases of pemphigoid gestationis were observed. All these cases occurred during the third trimester of pregnancy. Two participants had more than one specific dermatosis in pregnancy. The distribution of specific dermatoses of pregnancy is presented in Table 2.

**Table 2:** Distribution of specific dermatoses of pregnancy n: absolute count, %: percentage, G1: primigravida, G2: second gravida, G3: third gravida, G4: fourth gravida, PUPPP: pruritic urticarial papules and plaques of pregnancy

Specific dermatoses of pregnancy	First trimester				Second trimester				Third trimester				n (%)
	G1	G2	G3	G4	G1	G2	G3	G4	G1	G2	G3	G4	
	n (%)	n (%)	n (%)	n (%)	n (%)	n (%)	n (%)	n (%)	n (%)	n (%)	n (%)	n (%)	
Prurigo of pregnancy	0 (0.0%)	0 (0.0%)	0 (0.0%)	0 (0.0%)	0 (0.0%)	0 (0.0%)	0 (0.0%)	0 (0.0%)	12 (8.7%)	5 (3.6%)	0 (0.0%)	0 (0.0%)	17 (12.3%)
PUPPP	0 (0.0%)	0 (0.0%)	0 (0.0%)	0 (0.0%)	0 (0.0%)	0 (0.0%)	0 (0.0%)	0 (0.0%)	10 (7.2%)	1 (0.7%)	0 (0.0%)	0 (0.0%)	11 (7.9%)
Pruritus gravidarum	0 (0.0%)	0 (0.0%)	0 (0.0%)	0 (0.0%)	0 (0.0%)	0 (0.0%)	0 (0.0%)	0 (0.0%)	4 (2.8%)	2 (1.4%)	0 (0.0%)	0 (0.0%)	6 (4.3%)
Pruritic folliculitis	0 (0.0%)	0 (0.0%)	0 (0.0%)	0 (0.0%)	0 (0.0%)	0 (0.0%)	0 (0.0%)	0 (0.0%)	2 (1.4%)	0 (0.0%)	0 (0.0%)	0 (0.0%)	2 (1.4%)
Total	0 (0.0%)	0 (0.0%)	0 (0.0%)	0 (0.0%)	0 (0.0%)	0 (0.0%)	0 (0.0%)	0 (0.0%)	26 (18.8%)	8 (5.8%)	0 (0.0%)	0 (0.0%)	34 (24.6%)

Non-specific dermatoses were observed in 41 (29.7%) participants, and their distribution is presented in Table 3. Infectious dermatoses (active infections diagnosed during pregnancy) were the most common, noted in 30 (21.7%) participants. Bacterial infections were observed in six (4.3%) participants, and furunculosis was the most common bacterial infection. Out of nine (6.5%) participants with viral infections, varicella-zoster was seen in four (2.8%). Twenty (14.4%) participants had fungal infections, out of which candidiasis, tinea corporis, and tinea versicolor were observed in 12 (8.6%), six (4.3%), and two (1.4%) participants, respectively. Autoimmune dermatoses were less common and observed in four (2.8%) participants. This included SLE in two (1.4%), vitiligo in one (0.7%), and systemic sclerosis in one (0.7%) participant, respectively. Other miscellaneous conditions, such as acne, atopic dermatitis, psoriasis, seborrheic dermatitis, and pityriasis rosea, were observed in six (4.3%), three (2.1%), two (1.4%), two (1.4%), and one (0.7%) participants, respectively. Seven participants had more than one condition.

**Table 3:** Distribution of non-specific dermatoses of pregnancy n: absolute count, %: percentage, G1: primigravida, G2: second gravida, G3: third gravida, G4: fourth gravida, SLE: Systemic lupus erythematosus

	First trimester				Second trimester				Third trimester				n (%)
	G 1	G 2	G 3	G 4	G 1	G2	G3	G 4	G1	G2	G3	G 4	
	n (%)	n (%)	n (%)	n (%)	n (%)	n (%)	n (%)	n (%)	n (%)	n (%)	n (%)	n (%)	
Infectious condition													30 (21.7%)
Bacterial infection													6 (4.3%)
Furunculosis	0 (0.0%)	0 (0.0%)	0 (0.0%)	0 (0.0%)	1 (0.7%)	0 (0.0%)	0 (0.0%)	0 (0.0%)	2(1.4 %)	1 (0.7%)	0 (0.0%)	0 (0.0%)	4 (2.8%)
Hansen’s disease	1 (0.7%)	0 (0.0%)	0 (0.0%)	0 (0.0%)	1 (0.7%)	0 (0.0%)	0 (0.0%)	0 (0.0%)	0 (0.0%)	0 (0.0%)	0 (0.0%)	0 (0.0%)	2 (1.4%)
Viral infection													9 (6.5%)
Varicella zoster	0 (0.0%)	0 (0.0%)	0 (0.0%)	0 (0.0%)	1 (0.7%)	1 (0.7%)	0 (0.0%)	0 (0.0%)	2 (1.4%)	0 (0.0%)	0 (0.0%)	0 (0.0%)	4 (2.8%)
Condyloma accuminata	0 (0.0%)	0 (0.0%)	0 (0.0%)	0 (0.0%)	0 (0.0%)	0 (0.0%)	0 (0.0%)	0 (0.0%)	1 (0.7%)	0 (0.0%)	0 (0.0%)	0 (0.0%)	1 (0.7%)
Herpes simplex virus	0 (0.0%)	0 (0.0%)	0 (0.0%)	0 (0.0%)	1 (0.7%)	0 (0.0%)	0 (0.0%)	0 (0.0%)	1 (0.7%)	0 (0.0%)	0 (0.0%)	0 (0.0%)	2 (1.4%)
Herpes zoster	0 (0.0%)	0 (0.0%)	0 (0.0%)	0 (0.0%)	0 (0.0%)	0 (0.0%)	0 (0.0%)	0 (0.0%)	2 (1.4%)	0 (0.0%)	0 (0.0%)	0 (0.0%)	2 (1.4%)
Fungal infection													20 (14.4%)
Candidiasis	1 (0.7%)	0 (0.0%)	0 (0.0%)	0 (0.0%)	4 (2.8%)	1 (0.7%)	0 (0.0%)	0 (0.0%)	4 (2.8%)	1 (0.7%)	1 (0.7%)	0 (0.0%)	12 (8.6%)
Tinea corporis	0 (0.0%)	0 (0.0%)	0 (0.0%)	0 (0.0%)	2 (1.4%)	1 (0.7%)	0 (0.0%)	0 (0.0%)	2 (1.4%)	0 (0.0%)	1 (0.7%)	0 (0.0%)	6 (4.3%)
Tinea versicolor	1 (0.7%)	0 (0.0%)	0 (0.0%)	0 (0.0%)	0 (0.0%)	0 (0.0%)	0 (0.0%)	0 (0.0%)	1 (0.7%)	0 (0.0%)	0 (0.0%)	0 (0.0%)	2 (1.4%)
Protozoal infection													2 (1.4%)
Trichomoniasis	1 (0.7%)	0 (0.0%)	0 (0.0%)	0 (0.0%)	0 (0.0%)	1 (0.7%)	0 (0.0%)	0 (0.0%)	0 (0.0%)	0 (0.0%)	0 (0.0%)	0 (0.0%)	2 (1.4%)
Arthropod infection													2 (1.4%)
Scabies	1 (0.7%)	0 (0.0%)	0 (0.0%)	0 (0.0%)	0 (0.0%)	1 (0.7%)	0 (0.0%)	0 (0.0%)	0 (0.0%)	0 (0.0%)	0 (0.0%)	0 (0.0%)	2 (1.4%)
Autoimmune condition													4 (2.8%)
SLE	0 (0.0%)	0 (0.0%)	0 (0.0%)	0 (0.0%)	1 (0.7%)	0 (0.0%)	0 (0.0%)	0 (0.0%)	0 (0.0%)	1 (0.7%)	0 (0.0%)	0 (0.0%)	2 (1.4%)
Vitiligo	0 (0.0%)	0 (0.0%)	0 (0.0%)	0 (0.0%)	0 (0.0%)	0 (0.0%)	1 (0.7%)	0 (0.0%)	0 (0.0%)	0 (0.0%)	0 (0.0%)	0 (0.0%)	1 (0.7%)
Systemic sclerosis	0 (0.0%)	1 (0.7%)	0 (0.0%)	0 (0.0%)	0 (0.0%)	0 (0.0%)	0 (0.0%)	0 (0.0%)	0 (0.0%)	0 (0.0%)	0 (0.0%)	0 (0.0%)	1 (0.7%)
Miscellaneous													14 (10.1%)
Acne	0 (0.0%)	0 (0.0%)	0 (0.0%)	0 (0.0%)	2 (1.4%)	0 (0.0%)	0 (0.0%)	0 (0.0%)	2 (1.4%)	2 (1.4%)	0 (0.0%)	0 (0.0%)	6 (4.3%)
Atopic dermatitis	2 (1.4%)	0 (0.0%)	0 (0.0%)	0 (0.0%)	0 (0.0%)	1 (0.7%)	0 (0.0%)	0 (0.0%)	0 (0.0%)	0 (0.0%)	0 (0.0%)	0 (0.0%)	3 (2.1%)
Psoriasis	0 (0.0%)	0 (0.0%)	0 (0.0%)	0 (0.0%)	1 (0.7%)	0 (0.0%)	0 (0.0%)	0 (0.0%)	0 (0.0%)	0 (0.0%)	1 (0.7%)	0 (0.0%)	2 (1.4%)
Seborrheic dermatitis	0 (0.0%)	0 (0.0%)	0 (0.0%)	0 (0.0%)	0 (0.0%)	0 (0.0%)	0 (0.0%)	0 (0.0%)	0 (0.0%)	2 (1.4%)	0 (0.0%)	0 (0.0%)	2 (1.4%)
Pityriasis rosea	0 (0.0%)	0 (0.0%)	0 (0.0%)	0 (0.0%)	0 (0.0%)	1 (0.7%)	0 (0.0%)	0 (0.0%)	0 (0.0%)	0 (0.0%)	0 (0.0%)	0 (0.0%)	1 (0.7%)
Total													41 (28.7%)

When we analyzed the association between dermatoses in pregnancy and the gravida of the participants, we observed that primigravida had a significantly higher frequency of pigmentary changes (p = 0.027) and striae gravidarum (p = 0.012). However, the primigravida had a significantly lower frequency of vascular changes (p = 0.0001). The specific dermatoses of pregnancy were not statistically different between the groups (p = 0.050). The association between dermatoses in pregnancy and the gravida is shown in Table 4.

**Table 4:** Association between dermatoses in pregnancy and the gravida n: absolute count, %: percentage, df: degrees of freedom, *: Fisher's exact test

	Primigravida (n = 86)	Multigravida (n = 52)	Pearson’s chi-square	df	p-value
Pigmentary changes					
Yes	84 (97.7%)	45 (86.5%)	-	-	0.027*
No	2 (2.3%)	7 (13.5%)	-	-	-
Vascular changes					
Yes	4 (4.7%)	26 (50.0%)	39.171	1	<0.001
No	82 (95.3%)	26 (50.0%)	-	-	-
Striae gravidarum					
Yes	71 (82.6%)	33 (63.5%)	6.365	1	0.012
No	15 (17.4%)	19 (36.5%)	-	-	-
Specific dermatoses of pregnancy					
Yes	26 (30.2%)	8 (15.4%)	3.848	1	0.050
No	60 (69.8%)	44 (84.6%)	-	-	-

When we analyzed the association between dermatoses in pregnancy with the trimester of the participants, we observed that there was no significant difference in the pigmentary changes of participants from the three trimesters (p = 0.113). Participants in the third trimester had higher vascular changes (p = 0.001), striae gravidarum (p < 0.001), and specific dermatoses of pregnancy (p < 0.001). The association between dermatoses in pregnancy and the different trimesters is shown in Table 5.

**Table 5:** Association between dermatosis in pregnancy and the different trimesters n: absolute count, %: percentage

	First trimester (n = 6)	Second trimester (n = 56)	Third trimester (n = 76)	Fisher's exact test p-value
Pigmentary changes				
Yes	5 (83.3%)	55 (98.2%)	69 (90.8%)	0.113
No	1 (16.7%)	1 (1.8%)	7 (9.2%)	-
Vascular changes				
Yes	2 (33.3%)	4 (7.1%)	24 (31.6%)	0.001
No	4 (66.7%)	52 (92.9%)	52 (68.4%)	-
Striae gravidarum				
Yes	3 (50.0%)	30 (53.6%)	71 (93.4%)	<0.001
No	3 (50.0%)	26 (46.4%)	5 (6.6%)	-
Specific dermatoses of pregnancy				
Yes	0 (0.0%)	0 (0.0%)	34 (44.7%)	<0.001
No	6 (100.0%)	56 (100.0%)	42 (55.3%)	-

## Discussion

In this study, the majority (n = 86, 62.3%) of the participants were primigravida. Most women were in the third trimester (n = 76, 55.1%). We observed physiological skin changes in 136 (98.5%) participants (Table 1). Kumari et al. [21] and Panicker et al. [22] reported physiological skin changes in pregnancy in 100% and 99% participants, respectively. Among these physiological changes, we observed that pigmentary changes were most frequent, being present in 129 (93.4%) participants. Kumari et al. [21] and Panicker et al. [22] also reported pigmentary changes as the most common physiological change during pregnancy.

In this study, the most frequently observed pigmentary changes observed was linea nigra (118, 85.50%), followed by secondary areolar pigmentation (103, 74.63%) (Table 1). Kumari et al. [21] reported linea nigra and secondary areolar pigmentation in 91.4% and 78.4% of participants. Melasma was the third most frequent pigmentary change noted in our study, observed in 58 (42.02%) participants. Muzaffar et al. [23] reported melasma in 46.4% pregnant women. The high prevalence of pigmentary changes during pregnancy is attributed to hormonal fluctuations, particularly increased levels of melanocyte-stimulating hormone (MSH) and estrogen [24]. In our study, we observed that primigravida had a significantly higher frequency of pigmentary changes (p = 0.027) (Table 4). We also observed that there was no significant statistical difference in the pigmentary changes of participants from the three trimesters (p = 0.113) (Table 5). Kannambal and Tharini [20] also reported that primigravida had a higher frequency of pigmentary changes (p ≤ 0.001); however, they reported no significant statistical difference in the pigmentary changes of participants, trimester-wise (p = 0.349).

Striae gravidarum were observed in 104 (75.4%) participants in our study (Table 1). Previous studies by Kannambal and Tharini [20], Kumari et al. [21], and Panicker et al. [22] reported the frequency of striae gravidarum to be 79.7%, 72.8 %, and 79.6 %, respectively. Striae gravidarum emerged as the second most common physiological change observed during pregnancy in our study. Striae gravidarum demonstrated a significant association with both gravidity and trimester, being most frequently observed among primigravida (p = 0.012) and during the third trimester (p < 0.001) (Tables 4-5). Development of striae is mainly due to hormones like estrogen, relaxin, and adrenocortical hormones, which decrease the adhesiveness between collagen fibers and ground substances, along with stretching of the skin secondary to increasing abdominal girth during pregnancy [24].

In our study, vascular changes were observed in 30 (21.7%) participants (Table 1). Muzaffar et al. [23] reported vascular changes in 34.2% pregnant women. Kannambal and Tharini [20] reported vascular changes in 23.60% women. Among the vascular changes, pedal edema was the most frequent, being reported in 24 (17.39%) participants in our study. This finding is comparable to that reported by Kannambal and Tharini [20], where pedal edema was identified as the most common vascular alteration, occurring in 16.4% of cases. We observed that the occurrence of vascular changes was significantly higher in multigravida (p < 0.001) and those in their last trimester (p = 0.001). This increased frequency may be attributed to rising venous pressure in the femoral and pelvic vessels caused by compression from the gravid uterus [25].

We observed specific dermatoses of pregnancy in 34 (24.6%) participants (Table 2). Kumari et al. [21] and Panicker et al. [22] reported specific dermatoses of pregnancy in 3.6% and 22% of pregnant women, respectively. Prurigo of pregnancy was the most common specific dermatosis observed in our study, being present in 17 (12.3%) participants, which is consistent with the findings of Hassan et al. [25]. This may be due to population characteristics, regional variations, or classification differences. However, studies conducted by Kumari et al. [21] and Panicker et al. [22] identified PUPPP as the most common dermatosis of pregnancy. In our study, PUPPP was observed in 11 (7.9%) participants. PUPPP is linked with abdominal distention, which is more commonly observed in twin pregnancy and in the third trimester. This aligns with the hypothesis that rapid abdominal wall distension induces collagen antigens, which trigger an inflammatory lymphohistiocytic reaction, leading to connective tissue damage [13]. In this study, we observed that specific dermatoses of pregnancy were higher in primigravida, but this was not statistically significant (p = 0.050) (Table 4). However, Kannambal and Tharini [20] reported that specific dermatoses of pregnancy were significantly higher in primigravida (p < 0.0001).

In our study, among the nonspecific dermatoses of pregnancy, infections were the most commonly observed condition, occurring in 30 (21.7%) participants. Kannambal and Tharini [20], in their study, reported infections in 30.8% of pregnant women. In this study, we observed that fungal infections were the most frequent, occurring in 20 (14.4%) participants. Similar findings were reported by Kumari et al. [21] had also reported fungal infections as the most common infectious dermatosis during pregnancy. Among fungal infections, candidiasis was the most frequently encountered, affecting 12 (8.6%) participants. Similar findings were also reported by Kumari et al. [21] and Panicker et al. [22]. During pregnancy, glycogen-rich desquamated vaginal epithelial cells lower vaginal acidity, creating a favorable environment for *Candida* growth. It is hypothesized that elevated estrogen levels and higher glycogen content in vaginal secretions during the pregnancy period increase a woman's risk of developing vulvovaginal candidiasis [26]. Eastern India has a warm and humid climate, which increases the chances of acquiring fungal infections, particularly candidiasis, which explains the proportion of infectious dermatoses. In addition, low socioeconomic status, overcrowding, and limited access to healthcare facilities may also contribute to differences in disease prevalence.

The main strength of the study is that it represents the dermatoses in pregnant women from the eastern part of India, a region where insufficient data is available. All patients were evaluated by consultant dermatologists in a tertiary care center. Other strengths include the facilities for laboratory confirmation of suspected infections. Our study was not without limitations. A sample of 138 women may not be representative of the entire pregnant population. As this is a single-center tertiary care study, the findings may not be generalized to the entire population of pregnant women. The cross-sectional nature of the study made it impractical to determine causal relationships between pregnancy and dermatoses. Some skin conditions appear or resolve at different gestational stages, but a cross-sectional study does not track progression over time. A few women may not report mild or transient skin conditions, which may increase the chances of underestimation of certain dermatoses. This study does not assess persistence, resolution, or recurrence of skin conditions post-pregnancy. The effect of dermatoses on maternal or fetal health outcomes was not evaluated. Only six participants were enrolled in the first trimester, which might have reduced the statistical power of trimester-wise comparisons. There are chances of referral bias, hospital-based bias, and spectrum bias in our study findings. We also could not carry out a multivariable analysis.

## Conclusions

This study highlights the spectrum of physiological, specific, and non-specific dermatoses of pregnancy and their association with gravidity and trimester. Physiological changes, particularly pigmentary changes and striae gravidarum, were the most common findings. Specific dermatoses such as prurigo of pregnancy and PUPPP were more frequent in primigravida and later trimesters. In infective dermatoses, fungal infection, especially candidiasis, constitutes the major non-specific dermatoses of pregnancy. Physiological skin changes largely require reassurance alone. Prompt identification and management of pregnancy-specific and infective dermatoses remain essential for optimal maternal and fetal outcomes. We recommend that future researchers conduct multicentric prospective studies on this area to determine maternal-fetal implications of dermatoses in pregnancy.
